# Stages of pregnancy and weaning influence the gut microbiota diversity and function in sows

**DOI:** 10.1111/jam.14344

**Published:** 2019-07-01

**Authors:** Y.J. Ji, H. Li, P.F. Xie, Z.H. Li, H.W. Li, Y.L. Yin, F. Blachier, X.F. Kong

**Affiliations:** ^1^ Key Laboratory of Agro‐ecological Processes in Subtropical Region, Hunan Provincial Key Laboratory of Animal Nutritional Physiology and Metabolic Process, National Engineering Laboratory for Pollution Control and Waste Utilization in Livestock and Poultry Production Institute of Subtropical Agriculture, Chinese Academy of Sciences, Changsha Hunan China; ^2^ Institute of Occupational Health and Environmental Health, School of Public Health Lanzhou University Lanzhou China; ^3^ UMR PNCA AgroParisTech, INRA, Université Paris‐Saclay Paris France

**Keywords:** gut microbiota, metabolite, microbial diversity, sows, stages of pregnancy

## Abstract

**Aims:**

The gut microbiota is believed to play important roles in the health of pregnant mammals, including their nutrient metabolism, immune programming and metabolic regulation. However, until recently, the shifts in gut microbiota composition and faecal and blood metabolic activity during different stages of pregnancy had not been investigated.

**Methods and Results:**

We investigated the shifts in backfat thickness, plasma and faecal metabolites and gut microbiota on days 30, 60, 90 and 110 of pregnancy and on day 21 after parturition (weaning) in sows. The backfat thickness of sows did not significantly differ among the different stages of pregnancy. The plasma concentrations of lipid metabolites, including triacylglycerol (TG), total cholesterol, high‐density lipoprotein‐cholesterol, low‐density lipoprotein‐cholesterol and calcium were reduced (*P* < 0·05) during pregnancy. In addition, the concentration of these metabolites, except TG, reached their maximum at the time of weaning. We also found that Tenericutes, Fibrobacteres and Cyanobacteria varied significantly according to the stages of pregnancy in sows (*P* < 0·05). Most of the genera, such as *Clostridiales*, *Desulfovibrio*, *Mogibacteriaceae* and *Prevotella*, increased (*P* < 0·05) with the progression of pregnancy and decreased (*P* < 0·05) at weaning. The alpha diversity values (i.e., Shannon diversity and observed species) of sow gut microbiota increased (*P* < 0·05) from pregnancy to weaning. Pregnancy stages also significantly influenced (*P* < 0·05) the community structure (beta diversity) of gut microbiota. The progression of pregnancy was associated with changes in lipid metabolism and several carbohydrate‐degradation bacteria (i.e., *Prevotella*, *Succinivibrio*, *Bacteroides* and *Parabacteroides*).

**Conclusions:**

Although causal links between the measured parameters remain hypothetical, these findings suggest that the increased diversity and concentration of beneficial gut microbes are associated with the metabolism of pregnant sows.

**Significance and Impact of the Study:**

Manipulation of the sow gut microbiota composition may potentially influence metabolism and health during pregnancy.

## Introduction

Gastrointestinal bacterial communities are known to play critical roles in the functioning and health of their hosts, including their nutrient absorption, metabolism, immune programming and protection from pathogens (Dethlefsen *et al.* 2007). These bacterial communities are influenced by various host and environmental factors, such as host genetics (Goodrich *et al.* 2014), obesity (Ridaura *et al.* 2013), dietary intake (Wu *et al.* 2011), environmental parameters (Grzeskowiak *et al.* 2012), use of prebiotics, probiotics and antibiotics and pregnancy (Dethlefsen and Relman 2011; Debelius *et al.* 2016). The relationships between pregnancy and gut microbiota are of particular interest since pregnancy is characterized by dramatic changes in hormones, immune functions and metabolism to support the growth of the mother and foetoplacental unit (Newbern and Freemark 2011). The analysis of biological fluids, especially the blood, has been used to identify typical compounds to evaluate the underlying metabolic changes that occur during pregnancy (Shen *et al.* 2016). In addition, the maternal gut microbiota also undergoes dramatic changes throughout the gestation period (Kuperman and Koren 2016). Among the reported changes, changes in the amino acid and lipid content, energy metabolism, as well as gut microbiota composition and metabolic activity have been linked to pregnancy (Dai *et al.* 2015; DiGiulio *et al.* 2015; Mandal *et al.* 2016). The metabolic activity of the microbiota allows for the synthesis of various compounds, including short‐chain fatty acids (SCFA), indoles, ammonia, bioamines, gaseous compounds and vitamins (Blachier *et al.* 2017). These compounds, after intestinal absorption, can be modified by the host and may be actively involved in the host cells as co‐metabolites.

Recently, most studies have focused on changes that occur in the microbiota during pregnancy and have revealed an overall increase in Proteobacteria and Actinobacteria and a reduced diversity in the human gut (Koren *et al.* 2012). However, DiGiulio *et al. *(2015) showed that the taxonomic composition and diversity of the microbiota community remained remarkably stable in the vagina, distal gut and saliva during pregnancy. The exact role of intestinal microbiota and the balance of these microbes to the complex process of sustaining the conceptus *in utero* and in maintaining an adequate pregnant period is not yet known (Nelson *et al.* 2016). Several reports have evaluated the relationship between pregnancy and gut microbiota in humans, but few studies have explored the shifts in gut microbiota and other metabolites in sows throughout pregnancy until weaning.

Although previous studies have detected the ileal and colonic microbiota composition in Huanjiang mini‐pigs during pregnancy (Ji *et al.* 2016; Kong *et al.* 2016), such studies did not report the corresponding changes in plasma metabolites and the faecal microbiota in the pregnant and postpartum conditions. Therefore, in the present study, we investigated such factors in Yorkshire × Dutch Landrace sows. Pregnant sows were fed a restricted diet, in accordance with the practical production in order to avoid the unhealthy effects of obesity. The plasma and faeces metabolite composition and gut microbial composition were determined on days 30, 60, 90 and 110 of pregnancy and on day 21 after parturition (i.e., at weaning). We mainly addressed three questions: (i) How do the plasma and faeces metabolites change throughout the pregnancy and weaning stages? (ii) Do the alpha and beta diversity values of gut microbiota change during pregnancy and weaning? (iii) What are the relationships between gut microbiota and metabolic products? Our results have important significance for understanding the relationship between gut microbiota and pregnancy.

## Materials and methods

### Experimental design and ethical standards

This study was approved by the animal welfare committee of the Institute of Subtropical Agriculture, Chinese Academy of Sciences. The experiment was conducted between June and October 2016. In total, 40 Yorkshire × Dutch Landrace crossbred sows with second‐ or third‐parity (New Wellful Co. Ltd, Hunan, China) were used. After insemination, the sows were housed individually in crates (2·4 × 0·7 m) from day 1 to day 107 of pregnancy and then were housed in farrowing crates (2·2 × 1·8 m) until weaning. The sows were fed a commercial gestational diet (3100 kcal kg^−1^, DE) from mating until day 100 of pregnancy and received a commercial lactation diet (3300 kcal kg^−1^, DE) from day 101 of pregnancy to weaning at 21 days in the postfarrowing period. Diet formulations used in this study are presented in Table 1.

**Table 1:** Formulation and chemical composition of diets

Items	Gestational diet	Lactation diet
Ingredient, %		
Corn	58·67	60·46
Wheat bran	24·50	5·00
Ground wheat	–	2·00
Soybean oil	1·00	3·00
Soybean meal	11·50	19·50
Albumen powder	–	3·00
Imported fish	–	2·50
Lysine	0·12	0·30
Threonine	0·03	0·05
Antioxidants	0·02	0·03
Antimildew agent	0·06	0·06
Detoxification cagent	0·10	0·10
Premix*	4·00	4·00
Nutritional value^†^		
DE (MJ kg^−1^)	13·25	15·00
CP	14·00	18·00
Lys	0·75	1·10
Met	0·20	0·35
Thr	0·55	0·79

* Supplied the following amounts per kilogram of diet: vitamin A, 10 000 IU; vitamin D_3_, 2000 IU; vitamin E, 100 IU; vitamin K_3_, 2 mg; thiamine, 2 mg; riboflavin, 4 mg; nicotinic acid, 15 mg; d‐pantothenic acid, 10 mg; pyridoxine, 3 mg; d‐biotin, 0·2 mg; folic acid, 3 mg; vitamin B_12_, 0·02 mg; choline, 500 mg; Na (NaCl), 1·38 g; Fe (FeSO_4_·H_2_O), 80 mg; Cu (CuSO_4_·5H_2_O), 20 mg; Mn (MnSO_4_·H_2_O), 40 mg; Zn (ZnSO4·H_2_O), 100 mg; Co (CoCl), 0·1 mg; I (KI), 0·6 mg; and Se (Na_2_SeO_3_), 0·25 mg.

^†^ Calculated values.

The sows were fed 1·6 kg of the gestational diet after insemination, i.e., days 0 to 3 and then, fed 2·5 kg of the same diet according to the body condition of the pregnant sows from days 4 to 100. Sows received 3·5 kg of the lactation diet from days 101 to 113 and then received 2·5 kg on the day of parturition. Sows were fed twice per day (9:00 and 16:00) from insemination until 3 days after parturition. Thereafter, sows were gradually fed more until the feed was available ad libitum. From day 4 of lactation until weaning, sows were fed four times per day (9:00, 14:00, 18:00 and 22:00). Water was available ad libitum throughout the entire experiment.

### Sample collection

Blood (*n* = 8) and fresh faecal samples (*n* = 8) were collected on days 30, 60, 90 and 110 of the gestation period and on day 21 after parturition (weaning). At the same time, the backfat thickness (*n* = 20) was measured. A single blood sample was collected into heparinized tubes from each sow 2 h after feeding by jugular venipuncture using a 10 ml syringe. Plasma was collected after centrifugation for 10 min at 3000 ***g*** and 4°C and stored at −20°C. The fresh faecal samples were immediately collected after defecation and transported in a refrigerated container (−20°C).

### Measurements of carcass traits, plasma biochemical parameters and faecal metabolites

The backfat thickness (P_2_ site) was measured using a Renco lean‐meter (Renco Corporation, Golden Valley, MN). The concentrations of plasma metabolites, including total protein (TP), albumin (ALB), globulin (GLB), triacylglycerol (TG), total cholesterol (TC), high‐density lipoprotein‐cholesterol (HDL‐C), low‐density lipoprotein‐cholesterol (LDL‐C), calcium (Ca) and phosphorus (P) were analysed using a CX‐4 Automatic Biochemical Analyzer (Beckman Inc., Fullerton, CA) and commercial kits (Leadman Biochemistry Technology Company, Beijing, China) according to the manufacturers’ instructions. In addition, we also measured the activity (U l^−1^) of plasma enzymes, such as alanine aminotransferase (ALT), aspartate aminotransferase (AST), lactate dehydrogenase (LDH) and α‐amylase (α‐AMY). The faecal SCFAs, including straight‐chain fatty acids (acetate, propionate, butyrate, and pentanoate) and branched chain fatty acids (BCFA; isobutyrate and isopentanoate) were analysed using gas chromatography as described previously (Ji *et al.* 2018). The bioamines, including 1,7‐heptyl diamine, cadaverine, phenylethylamine, putrescine, tryptamine, tyramine, spermidine and spermine were measured using high‐performance liquid chromatography as described previously (Ji *et al.* 2018).

### DNA extraction and 16S rRNA gene sequencing

DNA extraction and 16S rRNA gene sequencing were conducted at the Environmental Genomic Platform of Chengdu Institute of Biology. The total microbial genomic DNA from faeces was extracted using a QIAamp DNA Stool Mini Kit (Qiagen, Hilden, Germany) according to the manufacturer's instructions. The DNA concentration of each sample was measured with a NanoDrop^®^ ND‐1000 instrument (NanoDrop Technologies Inc., Waltham, MA). The protocols of PCR amplification, gel extraction and sequencing library construction used in this study were described by Li *et al. *(2016). The extracted DNA was diluted to 10 ng µl^−1^ for the PCR amplifications. The universal primers: 515F (5′‐GTGYCAGCMGCCGCGGTA‐3′) and 909R (5′‐CCCCGYCAATTCMTTTRAGT‐3′), with a 12 nt unique barcode at 5′‐end of the 515F primer (Tamaki *et al.* 2011), were used to amplify the V4 region of microbial 16S rRNA gene. After PCR amplification, amplicons were extracted from 1·2 agarose gels and purified using the SanPrep DNA Gel Extraction Kit (Sangon Biotech, Shanghai, China) and quantified with a NanoDrop ND‐1000 instrument (NanoDrop Technologies Inc., Waltham, MA, USA). Purified amplicons of equimolar concentrations were then pooled together and paired‐end sequenced using an Illumina MiSeq sequencer (MiSeq Reagent Kit V.2,500 cycles) according to standard protocols (Caporaso *et al.* 2012).

### Bioinformatics analysis

Bioinformatics analyses followed previous study (Li *et al.* 2016). Briefly, the raw sequences were analysed using QIIME Pipeline‐ver. 1.7.0 (http://qiime.org/tutorials/tutorial.html). All sequences were trimmed and assigned to each sample based on their unique barcodes (barcode mismatches = 0). The overlapping paired‐end reads were merged using the flash‐1.2.8 software (Caporaso *et al.* 2012). The merged sequences with high quality (read length >300 bp, without an ambiguous base ‘N’ and an average base quality score >30) were used for further analysis. The aligned 16S rRNA gene sequences were used for a chimera check using the Uchime algorithm (Edgar *et al.* 2011). Operational taxonomic units (OTUs) were clustered at a 97% identity threshold using CD‐HIT (Li and Godzik 2006). Singleton OTUs were filtered out. Each sample was rarefied to the same number of reads (8467). The most abundant sequences within each OTU were designated as ‘representative sequences’ and aligned against the core set of Greengenes 13_8 reference database (DeSantis *et al.* 2006) using the PyNAST tool. The representative sequences were taxonomically classified using the Ribosomal Database Project classifier in the QIIME platform (Wang *et al.* 2007). The alpha diversity indices, including observed species and Shannon diversity, were calculated. The rarefaction curves of all samples were generated from the observed OTUs at the OTU level. To assess the beta diversity, the weighted UniFrac distance metrics, which use phylogenetic information to calculate community similarity (Lozupone and Knight 2005), were produced through the QIIME pipeline. Principal coordinates analysis (PCoA) plots of the dissimilarity metrics were also visualized using origin 8.5.

### Statistical analysis

An analysis of similarity (anosim) was used to reveal whether the beta diversity was significantly different across the pregnancy and parturition stages based on the weighted UniFrac matrices using the ‘vegan’ package in the R program (Li *et al.* 2017). The effects of the different stages on backfat thickness, plasma biochemical parameters, the apparent relative abundances of communities at the phylum and genus level and the alpha diversity indices were analysed using a one‐way analysis of variance with LSD *post hoc* test in SAS (SAS Institute, Inc., Cary, NC). *P*‐values <0·05 were considered to indicate statistical significance and *P*‐values: 0·05 ≤ *P* < 0·10 were considered to indicate a trend. Linear discriminant analysis effect size (LEfSe) was used to identify bacterial biomarkers at the different stages, based on *P*‐values < 0·05 and LDA scores >2·0. These analyses were performed online in the Galaxy workflow framework (http://huttenhower.sph.harvard.edu/galaxy/).

### Nucleotide sequence accession numbers

The original 16S rRNA gene data are available at the European Nucleotide Archive: accession no. PRJEB26887 (http://www.ebi.ac.uk/ena/data/view/PRJEB26887).

## Results

### Backfat thickness and plasma biochemical parameters

The mean backfat thickness of 20 sows were 21·32, 21·24, 20·72, 19·62, and 18·32 mm on days 30, 60, 90 and 110 of pregnancy and on day 21 after parturition (weaning). The backfat thickness of sows on days 30 and 60 of pregnancy were greater (*P* < 0·05) than at weaning.

The evaluations of plasma metabolites revealed that the pregnancy stages affected the metabolism of protein, fat, Ca and P (Table 2). Plasma ALB and P concentrations progressively increased throughout pregnancy, and were reduced at the time of weaning (*P* < 0·05). Conversely, the GLB, HDL‐C, LDL‐C, TC, TG and Ca concentrations were reduced (*P* < 0·05) during pregnancy. In addition, the concentrations of these plasma metabolites, except TG, reached their maximum levels at the time of weaning (*P* < 0·05). With regard to enzyme activity in the plasma, the ALT activity was the highest on day 110 of gestation, but the lowest at the time of weaning (*P* < 0·05). The AST activity was higher on days 90 and 110 of gestation and lower on the other days (*P* < 0·05). The α‐AMY activity was higher (*P* < 0·05) on days 30, 60 and 110 of pregnancy. The LDH activity did not differ significantly (*P*> 0·05) with the progression of pregancy.

**Table 2:** Plasma metabolite concentrations and enzyme activities of sows at different stages

Items	Days of gestation in sows				Weaning	SEM	*P*‐values
	30	60	90	110			
TP (g l^−1^)	71·03 ± 0·66^ab^	75·13 ± 2·25^a^	59·87 ± 2·10^b^	60·45 ± 2·37^b^	76·05 ± 5·58^a^	0·967	0·001
ALB (g l^−1^)	29·82 ± 0·64^b^	30·17 ± 1·58^b^	30·55 ± 1·51^b^	35·83 ± 0·86^a^	33·68 ± 0·57^ab^	0·584	0·002
GLB (g l^−1^)	41·22 ± 0·93^ab^	44·97 ± 1·98^a^	29·32 ± 2·61^bc^	24·62 ± 1·86^c^	42·37 ± 5·60^ab^	0·966	0·002
TG (mmol l^−1^)	0·25 ± 0·05^ab^	0·32 ± 0·04^a^	0·21 ± 0·03^ab^	0·12 ± 0·02^b^	0·14 ± 0·01^b^	0·106	0·004
TC (mmol l^−1^)	1·42 ± 0·06^abc^	1·47 ± 0·13^ab^	1·12 ± 0·04^bc^	1·03 ± 0·12^c^	1·65 ± 0·10^a^	0·172	0·001
HDL‐C (mmol l^−1^)	0·51 ± 0·02^bc^	0·51 ± 0·04^b^	0·41 ± 0·02^bc^	0·35 ± 0·03^c^	0·70 ± 0·07^a^	0·108	<0·001
LDL‐C (mmol l^−1^)	0·67 ± 0·04^ab^	0·74 ± 0·07^ab^	0·50 ± 0·04^b^	0·55 ± 0·07^ab^	0·77 ± 0·04^a^	0·129	0·006
Ca (mmol l^−1^)	2·41 ± 0·03^a^	2·48 ± 0·05^a^	2·37 ± 0·05^a^	2·18 ± 0·03^b^	2·52 ± 0·03^a^	0·108	<0·001
P (mmol l^−1^)	1·61 ± 0·03^c^	1·73 ± 0·06^bc^	1·96 ± 0·06^ab^	2·17 ± 0·08^a^	1·78 ± 0·09^bc^	0·143	<0·001
ALT (U l^−1^)	31·28 ± 1·89^ab^	30·50 ± 2·49^ab^	25·40 ± 2·14^b^	36·38 ± 1·52^a^	24·50 ± 1·97^b^	0·788	0·002
AST (U l^−1^)	19·08 ± 1·44^b^	18·78 ± 0·84^b^	31·02 ± 9·21^a^	37·45 ± 4·17^a^	23·30 ± 2·36^ab^	0·001	<0·001
LDH (U l^−1^)	193·17 ± 16·74	198·15 ± 10·88	256·73 ± 54·98	245·98 ± 20·64	212·70 ± 33·56	3·108	0·524
α‐AMY (U l^−1^)	3508·28 ± 336·53^a^	3240·07 ± 605·66^a^	1880·48 ± 398·75^b^	3467·73 ± 190·30^a^	1907·57 ± 476·36^b^	11·404	0·014

The days 30, 60, 90 and 110 are after artificial fertilization and weaning (day 21) is after parturition. The results are obtained from five independent experiments involving 40 sows. Significant difference is indicated by different letters.

The concentrations of almost all the SCFAs and bioamines, except 1,7‐heptyl diamine, differed significantly among the different stages of pregnancy (Table 3). Notably, we found that acetate, propionate, butyrate, the total SCFAs and total SCFAs decreased from days 30 to 110 of pregnancy, but suddenly increased at weaning (*P* < 0·05).

**Table 3:** The faecal concentrations of short‐chain fatty acids (SCFAs) and bioamines in sows at different stages

Items	Days of gestation in sows				Weaning	SEM	*P*‐values
	30	60	90	110			
SCFAs (mg g^−1^)							
Acetate	5·84 ± 0·50^b^	5·39 ± 0·20^b^	4·04 ± 0·27^c^	2·67 ± 0·44^d^	7·19 ± 0·48^a^	0·332	<0·001
Propionate	2·50 ± 0·13^b^	2·41 ± 0·07^b^	2·11 ± 0·13^c^	1·33 ± 0·06^d^	2·86 ± 0·11^a^	0·104	<0·001
Isobutyrate	0·27 ± 0·03^ab^	0·35 ± 0·04^a^	0·22 ± 0·04^b^	0·22 ± 0·02^b^	0·33 ± 0·01^a^	0·017	0·01
Butyrate	1·71 ± 0·09^a^	1·68 ± 0·15^a^	1·05 ± 0·12^b^	0·70 ± 0·06^b^	1·61 ± 0·16^a^	0·091	<0·001
Isovalerate	0·58 ± 0·04^a^	0·55 ± 0·05^a^	0·34 ± 0·04^b^	0·58 ± 0·05^a^	0·67 ± 0·04^a^	0·028	<0·001
Valerate	0·36 ± 0·06^a^	0·41 ± 0·04^a^	0·14 ± 0·03^b^	0·24 ± 0·02^b^	0·36 ± 0·01^a^	0·025	<0·001
Total BCFA	0·84 ± 0·06^b^	0·90 ± 0·07^b^	0·55 ± 0·05^c^	0·80 ± 0·07^d^	1·01 ± 0·06^a^	0·038	<0·001
Total straight‐chain fatty acids	10·40 ± 0·69^ab^	9·90 ± 0·28^ab^	7·33 ± 0·43^c^	5·45 ± 0·49^b^	12·04 ± 0·70^a^	0·489	<0·001
Total SCFA	11·24 ± 0·71^b^	10·80 ± 0·34^b^	7·88 ± 0·48^c^	6·25 ± 0·44^c^	13·05 ± 0·71^a^	0·473	<0·001
Bioamines (μg g^−1^)							
1,7‐heptyl diamine	0·73 ± 0·06^a^	0·81 ± 0·06^a^	0·84 ± 0·05^a^	0·67 ± 0·06^a^	0·87 ± 0·09^a^	0·031	0·228
Cadaverine	3·40 ± 0·46^a^	1·23 ± 0·21^c^	1·54 ± 0·12^bc^	2·78 ± 0·23^ab^	3·66 ± 0·87^a^	0·265	0·002
Phenylethylamine	7·21 ± 0·61^a^	3·70 ± 0·58^b^	6·80 ± 0·86^a^	6·08 ± 0·53^a^	5·63 ± 0·75^ab^	0·361	0·011
Putrescine	3·17 ± 0·23^b^	2·83 ± 0·25^b^	2·05 ± 0·29^b^	4·45 ± 0·37^a^	4·84 ± 0·74^a^	0·259	<0·001
Spermidine	7·53 ± 1·03^b^	5·05 ± 0·86^b^	5·01 ± 0·25^b^	14·46 ± 1·46^a^	6·20 ± 0·56^b^	0·76	<0·001
Spermine	1·25 ± 0·27^a^	0·59 ± 0·08^b^	0·73 ± 0·04^b^	1·46 ± 0·22^a^	0·54 ± 0·06^b^	0·097	0·001
Tryptamine	2·47 ± 0·50^a^	0·22 ± 0·05^c^	1·16 ± 0·26^b^	0·54 ± 0·19^bc^	0·70 ± 0·14^bc^	0·185	<0·001
Tyramine	1·13 ± 0·23^b^	0·47 ± 0·06^b^	0·84 ± 0·25^b^	1·08 ± 0·19^b^	2·92 ± 0·72^a^	0·219	0·001
Total bioamine	26·89 ± 1·72^ab^	14·89 ± 1·68^c^	18·99 ± 1·07^c^	31·52 ± 1·61^a^	25·36 ± 2·65^bc^	1·33	<0·001

The days 30, 60, 90 and 110 are after artificial fertilization and weaning (day 21) is after parturition. The results are obtained from five independent experiments involving 40 sows. Significant difference is indicated by different letters.

### Alpha diversity and beta diversity of gut microbiota

**Figure 1:**
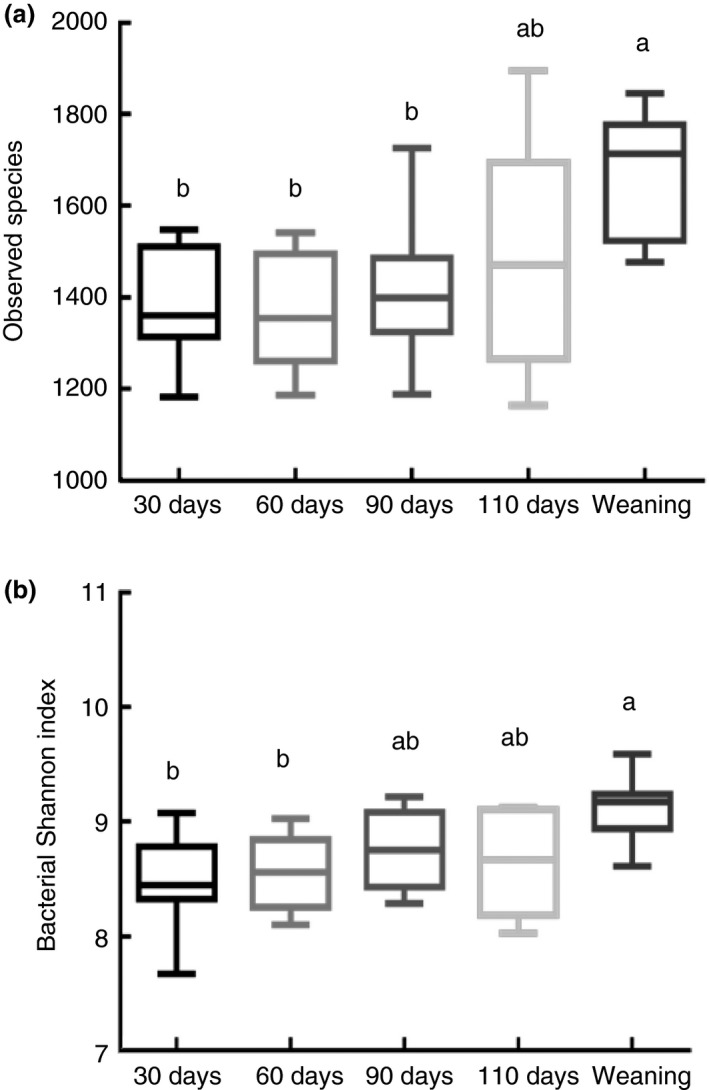
The changes of faecal microbial alpha diversity in different stages of sows. (a) The observed species of faecal bacteria in sows. (b) Shannon diversity index. Different letters above the bars denote a significantly different alpha diversity index among groups. The days 30, 60, 90 and 110 are after artificial fertilization and weaning (day 21) is after parturition. The results are obtained from five independent experiments involving 40 sows.

To compare samples with different sequencing depths, each sample was rarefied to 8467 reads. Based on 97% sequence similarity, all the sequences were clustered into 11 587 bacterial OTUs. The OTU‐level rarefaction curve of observed OTUs across all samples reached stable values (Fig. S1), indicating that the sampling depth provided sufficient OTU coverage to accurately describe the faecal bacterial diversity in this study. To further dissect the changes in the gut bacterial communities throughout pregnancy until weaning, the alpha diversity indices, including observed species (Fig. 1a) and Shannon index (Fig. 1b) were investigated. These indices increased with the progress of gestation and exhibited the largest values at the time of weaning (*P* < 0·05).

**Figure 2:**
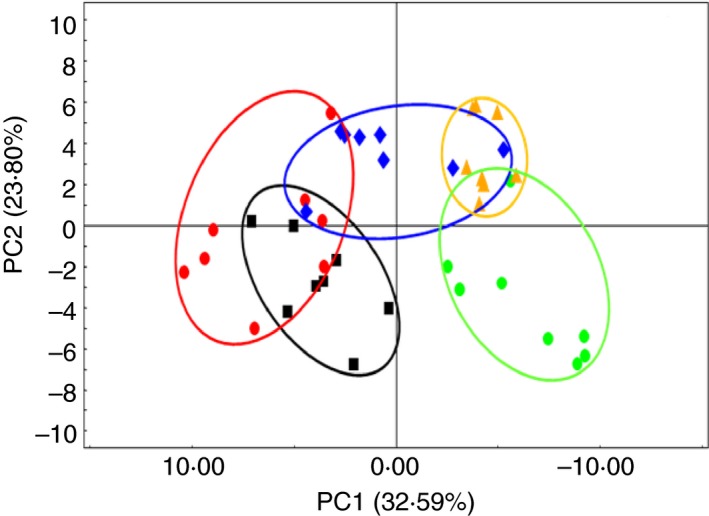
Scatterplot from PCoA of OTUs shown the differences in microbial community structures of sows among the five  physiological stages. The days (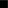) 30, (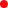) 60, (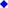) 90, and (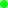) 110 are after artificial fertilization and (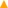) weaning (day 21) is after parturition. [Colour figure can be viewed at http://wileyonlinelibrary.com]

The PCoA based on the weighted UniFrac distance showed that the samples clustered together according to the stages of pregnancy and weaning, which indicated a shift in the gut bacterial community structure with the changes in the physiological state (Fig. 2). anosim analysis confirmed significant separation of gut bacterial communities among the different stages (*R* = 0·32, *P* < 0·001).

### Taxonomic composition in the gut microbiota from pregnancy to weaning

**Figure 3:**
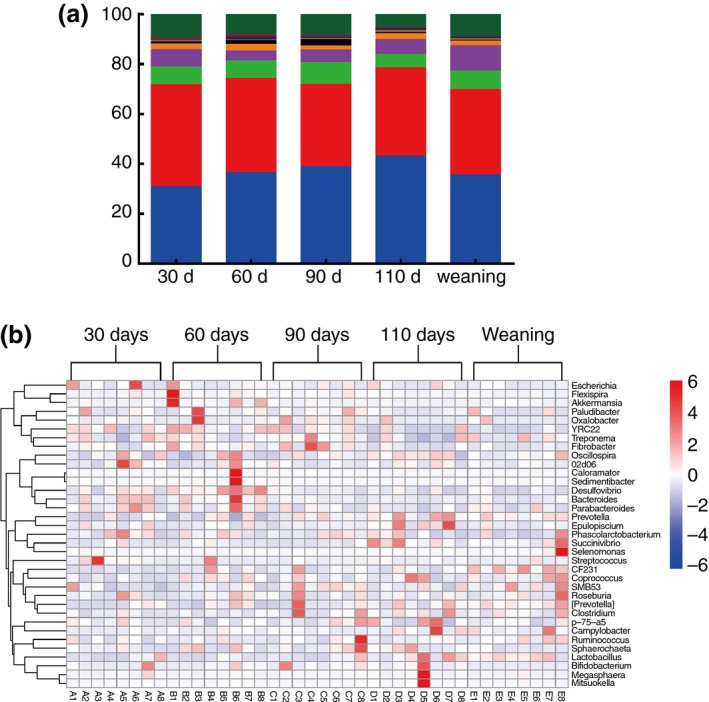
Taxonomic composition of faecal bacterial communities in different stages in sows. (a) Phylum‐level taxonomic composition of faecal bacterial communities in different stages in sows (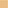) other bacteria (<0·5%); (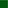) unclassified bacteria;(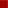) Actinobacteria; (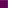) Verrucomicrobia; (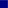) Planctomycetes; (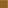) Cyanobacteria; (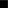) Fibrobacteres; (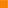) Proteobacteria; (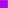) Tenericutes; (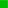) Spirochaetes; (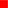) Firmicutes; (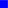) Bacteroidetes. (b) Heat map of genera in the relative abundances of sows faecal bacterial communities in five stages (30 d, 30 days of pregnancy; 60 d, 60 days of pregnancy; 90 d, 90 days of pregnancy; 110 d, 110 days of pregnancy; weaning, 21 days after parturition). [Colour figure can be viewed at http://wileyonlinelibrary.com]

In total, 23 phyla were identified within the faecal microbiota. The abundances of the 10 most abundant phyla (>0·5%) in each group are shown in Fig. 3a. Other phyla included: Acidobacteria, Chlamydiae, Chloroflexi, Deferribacteres, Elusimicrobia, Fusobacteria, Gemmatimonadetes, Lentisphaerae, OP8, Synergistetes, Thermi, TM7 and WPS‐2. Among the 23 phyla, Bacteroidetes and Firmicutes were detected as the dominant phyla regardless of the pregnancy stages and their total proportions reached as high as >60%. However, their relative abundance differed among the stages of pregnancy and weaning. Bacteroidetes increased linearly (*P* = 0·053) with the progression of pregnancy; becoming dominant on days 90 and 110 (42·11 and 45·56% respectively) of pregnancy. However, the abundance of Bacteroidetes decreased (*P* < 0·05) at weaning (38·67%). The abundance of Tenericutes was the lowest on day 60 of pregnancy and the highest at weaning (*P* < 0·05). Fibrobacteres increased (*P* < 0·05) linearly from days 30 to 90 of pregnancy (0·90, 1·55 and 2·52% respectively), but decreased (*P* < 0·05) to the lowest abundance on day 110 of pregnancy and at weaning (0·44 and 0·39% respectively). Cyanobacteria were found at the lowest abundance (0·28%) on day 60, but comprised 1% of the microbiota on day 110 of pregnancy (*P* < 0·05).

To further investigate the taxonomic compositions of faecal samples, a total of 325 genera were identified. Among these genera, 35 had a mean relative abundance ≥0·5% of the total sequences in at least one sample (Fig. 3b). The cluster analysis based on a heat map demonstrated a higher similarity of the samples within groups than among groups. *Prevotella* (phylum: Bacteroidetes) was the most abundant genera in the faecal microbiota. The abundance of *Prevotella* linearly increased with the progression of pregnancy (i.e., represented 7·65, 7·51, 10·07 and 14·02% of the microbiota at 30, 60, 90 and 110 days of pregnancy respectively) and decreased at weaning (11·83%) (*P* < 0·05). The genus *YRC22* was the most abundant on day 60 of pregnancy (5·66%), and decreased to the lowest levels at weaning (1·24%) (*P* < 0·05). The genus *Bacteroides* reached its maximum abundance (peaked) on day 60 of pregnancy (2·17%) (*P* < 0·05). The genus *CF231* peaked at weaning (*P* < 0·05). Within the Firmicutes phylum, the genus *Lactobacillus* was more abundant on day 110 of pregnancy and at weaning (6·91% and 6·21% respectively) (*P* < 0·05). The genus *Epulopiscium* peaked on day 110 of pregnancy (*P* < 0·05). *Fibrobacter* (phylum: Fibrobacteres) peaked at 2·52% on day 90 of pregnancy (*P* < 0·05). In the Proteobacteria phylum, the genera *Desulfovibrio* and *Succinivibrio* peaked at 0·90 and 0·58% on days 60 and 110 of pregnancy respectively (*P* < 0·05). *Akkermansia* (phylum: Verrucomicrobia) peaked at 0·13% on day 60 of pregnancy (*P* < 0·05).

### Microbial biomarkers of different pregnant stages

**Figure 4:**
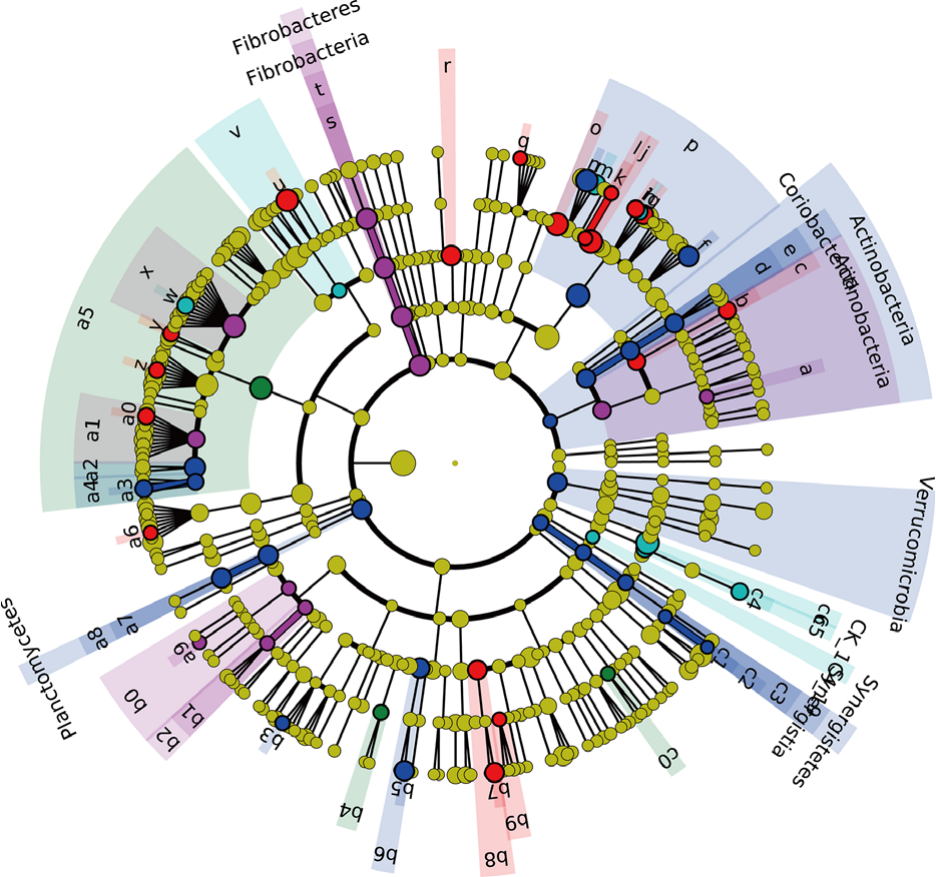
Cladogram plotted from LEfSe analysis indicated the biomarkers of the microbial community in different groups (*P* < 0·05; LDA score 2). (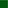) 30 d, 30 days of pregnancy; (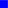) 60 d, 60 days of pregnancy; (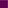) 90 d, 90 days of pregnancy; (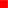) 110 d, 110 days of pregnancy; (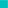) weaning, 21 days after parturition (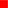) a: Bifidobacterium; (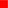) b: Bifidobacteriales; (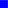) c: Coriobacteriaceae; (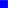) d: Coriobacteriales; (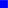) e: Bacteroides; (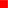) f: S24_7; (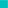) g: CF231; (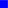) h: YRC22; (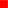) i: p_2534_18B5; (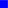) j: Bacteroidales; (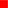) k: Mesonia; (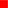) l: YS2; (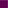) m: Fibrobacteraceae; (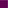) n: Fibrobacterales; (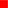) o: Lactobacillus; (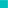) p: Lactobacillales; (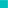) q: Lachnospira; (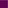) r: Lachnospiraceae; (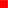) s: Peptococcus; (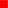) t: Faecalibacterium; (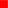) u: Anaerovibrio; (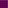) v: Veillonellaceae; (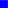) w: _Mogibacteriaceae_; (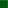) x: Clostridiales; (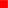) y: _Eubacterium_; (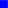) z: Pirellulaceae; (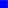) a0: Pirellulales; (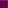) a1: Rhizobiales; (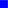) a2: Aquabacterium; (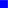) a3: Desulfovibrio; (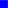) a4: Desulfovibrionales; (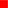) a5: Succinivibrio; (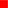) a6: Aeromonadales; (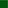) a7: Moraxellaceae; (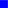) a8: Synergistales; (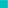) a9: Anaeroplasma). [Colour figure can be viewed at http://wileyonlinelibrary.com]

Linear discriminant analysis effect size analysis has been used to identify the biomarker species that distinguish the microbial communities at different stages. In our study, the dominant species from the faecal samples at 30 days of pregnancy were mainly from the classes Clostridiales and Moraxellaceae. The biomarker species at day 60 of pregnancy belonged to classes Coriobacteriaceae, Coriobacteriales, Bacteroides, YRC22, Bacteroidales, [Mogibacteriaceae], Pirellulaceae, Pirellulales, Aquabacterium, Desulfovibrio, Desulfovibrionales and Synergistales. The dominant species at day 90 of pregnancy were predominantly from the classes Fibrobacteraceae, Fibrobacterales, Lachnospiraceae, Veillonellaceae and Rhizobiales. The dominant species at day 110 of pregnancy were from the classes Bifidobacterium, Bifidobacteriales, S24‐7, p‐2534‐18B5, Mesonia, YS2, Lactobacillus, Peptococcus, Faecalibacterium, Anaerobibrio, [Eubacterium], Succinivibrio and Aeromonadales. The biomarker species at weaning were predominantly from the classes CF231, Lactobacillales, Lachnospira and Anaeroplasma (Fig. 4).

### Relationship between the bacterial community and bacterial metabolites

**Figure 5:**
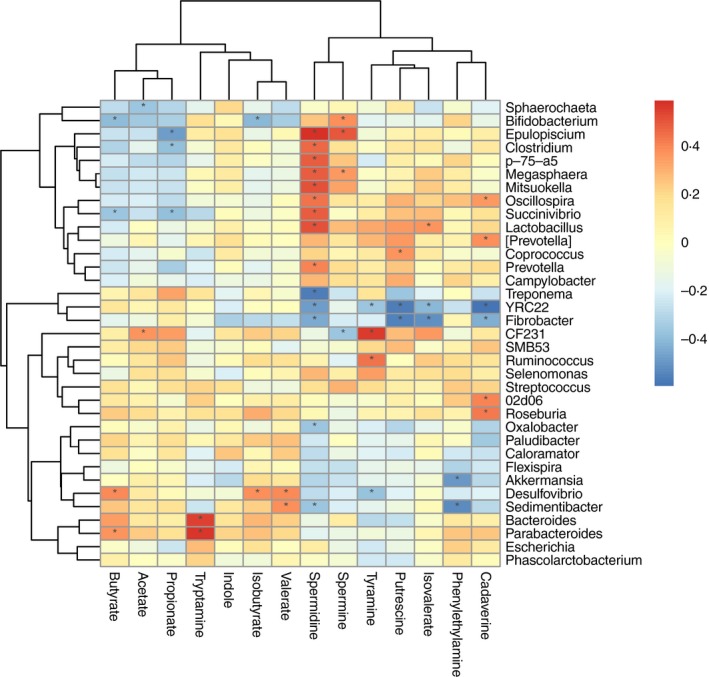
Correlations between the genera and the faecal metabolite concentrations. Only the genera for which abundance was significantly associated with the faecal concentrations of metabolites are presented; the red represents a significantly positive correlation, the blue represents a significantly negative correlation, and the white represents no significant correlation. * means the *P* value < 0.05. [Colour figure can be viewed at http://wileyonlinelibrary.com]

Particular genera were found to be linked to faecal metabolite concentrations (Fig. 5). The genera *Bacteroides* and *Parabacteroides* were positively correlated with tryptamine, while the genera *Akkermansia* and *Sedimentibacter* were negatively correlated with phenylethylamine; *YRC22* and *Fibrobacter* were also negatively, but *Coprococcus* was positively correlated with putrescine. Five genera were associated with cadaverine (positive correlations: [*Prevotella*], *02d06* and *Roseburia*; negative correlations: *YRC22* and *Fibrobacter*); four genera were associated with tyramine (positive correlations: *CF231* and *Ruminococcus*; negative correlations: *YRC22* and *Desulfovibrio*); 14 genera were associated with spermidine (positive correlations: *Prevotella*, *Lactobacillus*, *Oscillospira*, *p‐75‐a5*, *Clostridium*, *Succinivibrio*, *Megasphaera*, *Epulopiscium* and *Mitsuokella*; negative correlations: *Treponema*, *YRC22*, *Fibrobacter*, *Oxalobacter* and *Sedimentibacter*); three genera were associated with spermine (positive correlations: *Epulopiscium* and *Bifidobacterium*; negative correlation: *CF231*); genus *Parabacteroides* was positively, but *Sphaerochaeta* was negatively correlated with acetate; three genera *Clostridium*, *Succinivibrio* and *Epulopiscium* were negatively correlated with propionate; two genera were associated with isobutyrate (positive correlation: *Desulfovibrio*; negative correlation: *Bifidobacterium*); four genera were associated with butyrate (positive correlations: *Parabacteroides* and *Desulfovibrio*; negative correlations: *Succinivibrio* and *Bifidobacterium*); the genera *YRC22* and *Fibrobacter* were negatively, but *Lactobacillus* was positively correlated with isovalerate, while *Desulfovibrio* and *Sedimentibacter* were positively correlated with valerate. A total of 11 genera were associated with indole (positive correlations: *Prevotella*, *Lactobacillus*, *Oscillospira*, [*Prevotella*], *Succinivibrio*, *Campylobacter* and *Selenomonas*; negative correlations: *YRC22*, *Bacteroides*, *Parabacteroides* and *Desulfovibrio*).

## Discussion

This study investigated the shifts in plasma and faecal metabolites and in the gut microbiota of Yorkshire × Dutch Landrace sows during pregnancy (days 30, 60, 90 and 110 after artificial fertilization) and at weaning (day 21 after parturition). The results revealed significant metabolic changes in the maternal plasma and faeces at different stages, mainly involving the changes in lipid and protein metabolism, SCFAs and bioamines, as well as shifts in the gut microbiota composition in sows from pregnancy to weaning. Our results were consistent with those of Liu *et al. *(2018), who also demonstrated shifts in the gut microbiota and SCFA profiles of sows during pregnancy. These results may help to improve animal health during pregnancy via the manipulation of gut microbial communities.

Assessments of the blood biochemical parameters of animals can provide information on the metabolic state of the animals and help to improve the health status of the animals (Friendship 1992). The present study showed that plasma concentrations of GLB and TP were reduced on days 90 and 110 of pregnancy and recovered at weaning. As expected, protein digestion and absorption in the maternal hepatic and adipose tissues were likely to have been activated to preserve energy needs for the foetus (Shen *et al.* 2016). Previously, TP has been shown to be associated with GLB, but not with ALB. In addition, the GLB and TP in the serum of sows were found to decrease when nearing parturition (Machadoneto *et al.* 1987; Goff and Horst 1997; Castillo *et al.* 2005). Thus, the contents of TP and GLB in the blood are expected to be dependent on the nutritional needs of the foetus.

Maternal lipid metabolism plays a pivotal role in the initiation and development of pregnancy (Pinto *et al.* 2015) and the lipid concentrations are very sensitive to the energy balance (Shen *et al.* 2016). Pregnancy might affect lipid metabolism and thus lead to changes in the levels of many lipid compounds, including LDL, VLDL, TC, and TG. The present study showed that the plasma concentrations of HDL‐C, LDL‐C, TC and TG all decreased with the progression of pregnancy and recovered at weaning (except TG). We speculated that sows need to provision more nutrients for foetal development during pregnancy, so that no excess fat is used to synthesize blood lipids. Our results are consistent with those of Metges *et al*. (2012) found that the plasma concentrations of LDL‐C and HDL‐C increased at −5, 24, 66 and 108 days post coitum regardless of differences in diet (i.e., low protein, adequate protein or high protein diets), and that TC decreased linearly in sows fed the adequate protein diet. The serum cholesterol concentration decreased linearly from 14 days to 2 weeks prepartum (Tumbleson *et al.* 1970). These changes might be related to some oestrogen, such as progesterone. In a study by Saleri *et al. *(2015), the plasma progesterone levels were low, but increased to 10‐fold and then reached a plateau during the early stages of gestation. The progesterone levels then decreased from day 86 of pregnancy until day 10 after parturition and thereafter remained at lower levels (Saleri *et al.* 2015). These results indicated that blood hormone concentrations are closely dependent on the pregnancy and parturition stages in sows.

The present study showed an increased alpha diversity in the gut microbiota throughout the progression of pregnancy until weaning. Diversity is known to improve the stability and performance of communities and is, therefore, very important in many ecosystems (Margalef 1963; McNaughton 1977; Tap *et al.* 2015; Li *et al.* 2018). In particular, gut microbial diversity has been proposed as a new biomarker of health and metabolic capacity (Clarke *et al.* 2014). We speculate that a diverse gut microbiota probably provides many metabolic capacities and functional redundancy in sows, which ensures the sufficient supply of nutrients for foetal growth and development. However, further research is needed to investigate this inference. Our results of the anosim and PCoA analyses and the clustering of samples also suggested that the gut microbiota differed among the stages of pregnancy. It is noteworthy that our findings demonstrated that the structure and composition of the gut microbiota can vary considerably over time. As previously reported (Lu *et al.* 2014; Pajarillo *et al.* 2015), the phyla Bacteroidetes and Firmicutes were the most abundant in the gut of sows, regardless of the stage of pregnancy. Jost *et al. *(2014) also reported that Firmicutes exhibited no detectable changes over the perinatal period (Jost *et al.* 2014). However, the findings of our study differed from those of Koren *et al. *(2012) who reported an overall increase in Proteobacteria and Actinobacteria. The present study demonstrated that the abundance of Tenericutes, Fibrobacteres and Cyanobacteria changed significantly among the different physiological stages of sows. In terms of the phylum level, numerous studies have indicated that obesity and gestational weight gain are associated with an increase in the abundance of Firmicutes or an increase in the Firmicutes to Bacteriodetes ratio (Ley *et al.* 2006; Turnbaugh *et al.* 2009; Feng *et al.* 2015). It is noteworthy that the backfat thickness of sows did not differ greatly among the different stages of pregnancy in the present study. Due to the modulation of the diet, the sows did not gradually deposit more fat throughout the progression of pregnancy and the abundance of Firmicutes was maintained at a stable level in the gut. The Tenericutes phylum may provide some beneficial effects on the intestinal integrity because lower counts were detected in inflamed intestines, induced by dextran sodium sulphate (Nagalingam *et al.* 2011). The representatives of Fibrobacteres were characterized as having the potential to metabolize nonsoluble polysaccharides, such as cellulose, hemicellulose or pectin (Kubasova *et al.* 2017). Specifically, the genera *Prevotella* and *CF231* (phylum: Bacteroidetes), *Lactobacillus* (phylum: Firmicutes) and *Succinivibrio* (phylum: Proteobacteria) increased, but the genera *YRC22*, *Bacteroides* and *Parabacteroides* (phylum: Bacteroidetes); and *Desulfovibrio* (phylum: Proteobacteria) decreased from day 30 of pregnancy until weaning. Most of the genera increased with the progression of pregnancy and decreased at weaning. These included the genera *Prevotella* and *YRC22* and *S24‐7* and *p‐2534‐18B5* families within the phylum Bacteroidetes; the species *Fibrobacter succinogenes* within the phylum Fibrobacteres; the order Clostridiales and family Mogibacteriaceae within the phylum Firmicutes; the genera *Succinivibrio* and *Desulfovibrio* within the phylum Proteobacteria; the genus *Treponema* within the phylum Spirochaetes and the order *RF39* within Tenericutes. *Prevotella* is more common in humans who consume a plant‐rich diet (Ley 2016). In addition to *Prevotella*, *Succinivibrio* is a known plant polysaccharide‐fermenting bacteria in the *Prevotella‐*type gut microbial community of native Africans (Ou *et al.* 2013). *Bacteroides* was characterized by a high expression of xylose isomerase. In addition to *Bacteroides*, *Parabacteroides* is a producer of propionic acid as a metabolic end‐product (MacFabe *et al.* 2007). *Lactobacillus* as a lactic acid‐producing bacterium could  degrades lactose and other oligosaccharides into acetate and lactate (Walter 2008). Some members of *Desulfovibrio* can produce hydrogen sulphide by reducing sulphates (Heidelberg *et al.* 2004). By producing hydrogen sulphide, *Desulfovibrio* may, therefore, impact the metabolism of epithelial cells (Fite *et al.* 2004; Bisson‐Boutelliez *et al.* 2010). *Fibrobacter succinogenes* is an important degrader of lignocellulosic plant material (Bera‐Maillet *et al.* 2004). The order Clostridiales can digest protein, carbohydrate, sugar, amino acid, purine, pyrimidine and other organic compounds, to produce SCFAs (Zhang *et al.* 2016a). The genus *Treponema* comprises several uncultivable human and animal pathogens (Cejkova *et al.* 2015). As the LEfSe analysis showed in the present study, we also detected that the classes related to nutrient digestion increasing in the later stages of pregnancy included Fibrobacteraceae, Fibrobacterales, Bifidobacterium, Bifidobacteriales, Lactobacillus and Faecalibacterium*.* As previously mentioned, we observed numerous changes throughout the pregnancy of sows and most of the bacteria that increased in abundance with the progression of pregnancy are able to digest plant‐type materials, such as oligosaccharides, polysaccharides, lignocellulose and other cellulose‐type materials. This implies that undigestible or partly digestible dietary compounds can be metabolized by the intestinal microbiota for the potential benefit of the sows and their foetuses. The observed changes in the phylum and genera also revealed that most of the bacteria that showed increases during pregnancy, decreased at weaning. These findings showed that the changes in gut microbiota were transient at the critical period of pregnancy, but that the gut microbiota returned to a normal composition after this critical time. In addition to changes in the physiologies of animals, the changes in gut microbiota may also result from differences in dietary components between pregnancy and lactation stages. In a previous study, the diet of pregnant sows contained more wheat bran, which was demonstrated to influence pig gut microbial communities (Kraler *et al.* 2016).

As the major metabolites produced by gut microbiota, SCFAs have been suggested to be important regulators of energy balance, gut inflammation signalling and insulin sensitivity (Russell *et al.* 2011). Other compounds such as polyamines, other bioamines and indoles are produced by colonic bacteria from their respective amino acid derivities (Davila *et al.* 2013). According to the correlation analyses in the present study, a significant correlation was detected between the bacterial genera and metabolite concentrations. These results are consistent with those from a previous study that showed that bacteria with protein‐fermentation or AA‐fermentation capacities within the genera *Bacteroides*, *Parabacteroides*, *Lactobacillus*, *Megasphaera*, *Roseburia* and *Ruminococcus* (Davila *et al.* 2013; Dai *et al.* 2015; Zhang *et al.* 2016b) are positively correlated with bioamine concentrations. Some studies have shown that the genera *Akkermansia*, *Fibrobacter* and *Desulfovibrio*, i.e., are plant‐degradation bacteria (Fite *et al.* 2004; Hill and Spence 2017), as well as *Sedimentibacter*, *Treponema* and *YRC22* are negatively correlated with bioamine concentrations. Higher concentrations of SCFAs were associated with carbohydrate‐degradation bacteria. Although some bacterial genera were correlated with SCFAs, it remains difficult to predict which bacteria are responsible for the production of specific SCFAs due to the complexity of microbial interactions, such as cross‐feeding (Rey *et al.* 2010) and resource competition (Mahowald *et al.* 2009). However, these bacteria probably play important roles in the gut fermentation of food in sows.

Collectively, our findings suggest that the progression of pregnancy is associated with changes in lipid metabolism and in the composition of the intestinal microbiota; notably of the bacteria involved in the degradation of carbohydrates, such as the genera *Prevotella*, *Succinivibrio*, *Bacteroides*, *Parabacteroides* and the order Clostridiales. The changes in gut microbiota diversity were transient and notably related to some critical processes at different stages during pregnancy. The present study mainly explored the associations between microbiota composition and metabolic parameters in sows during pregnancy until weaning, but did not determine the possible causal links between these parameters. Therefore, future studies should shed light on the ecological and functional mechanisms of the changes in the gut microbiota throughout pregnancy until weaning to determine such potential links between the metabolic activity of the microbiota and the metabolic changes in the hosts.

## Author contributions

X.F.K., Y.J.J. and Y.L.Y. performed the experiments. Y.J.J., H.L., X.F.K. and F.B. wrote the manuscript. Y.J.J. and H.L. performed the statistical analyses. P.F.X., Z.H.L. and H.W.L. fed the animals. All authors reviewed the manuscript.

## Conflict of Interest

The authors declare no competing financial interests.

## Supporting information


**Figure S1**. The OTU‐level rarefaction curve of observed OTUs across all samples.
