# Supplementary figures and images for: Stages of pregnancy and weaning influence the gut microbiota diversity and function in sows
Source: J Appl Microbiol. 2019 Jul 1;127(3):867–79. doi: 10.1111/jam.14344 (PMC6852164; doi:10.1111/jam.14344)

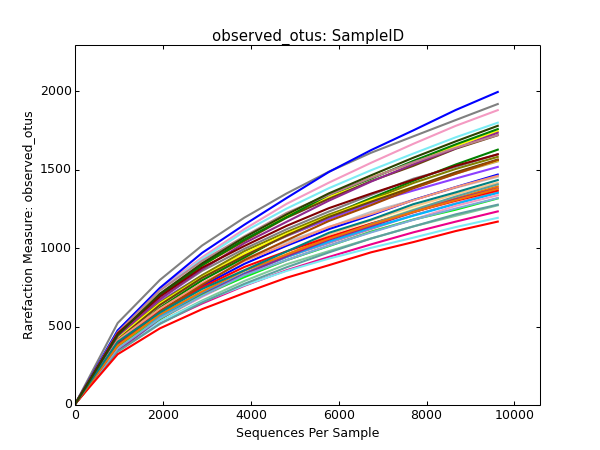


Figure S1. The OTU-level rarefaction curve of observed OTUs across all samples.

Supplement: Supplementary file 1 — Figure S1. The OTU‐level rarefaction curve of observed OTUs across all samples. [file JAM-127-867-s001.docx]
